# Feasibility and Safety of Repeated Intraperitoneal Platinum and Pegylated Liposomal Doxorubicin for First Recurrent Ovarian Cancer: A Preliminary Retrospective Comparative Study

**DOI:** 10.3390/cancers18152404

**Published:** 2026-07-25

**Authors:** Wan-Hua Ting, Hui-Hua Chen, Ta-Cheng Lee, Chih-Hsun Wang, Ming-Chow Wei, Sheng-Mou Hsiao

**Affiliations:** 1 Department of Obstetrics and Gynecology, Far Eastern Memorial Hospital, New Taipei 220216, Taiwan; stellatingwh@gmail.com (W.-H.T.); thandaaye24@gmail.com (H.-H.C.); s8282sn@gmail.com (T.-C.L.); taurus63119@gmail.com (C.-H.W.); wei@mail.femh.org.tw (M.-C.W.); 2 Department of Electrical Engineering, Yuan Ze University, Taoyuan 320315, Taiwan; 3 Department of Biomedical Engineering, School of Health and Medical Engineering, Ming Chuan University, Taoyuan 333321, Taiwan; 4 Department of Obstetrics and Gynecology, National Taiwan University College of Medicine, National Taiwan University Hospital, Taipei 100226, Taiwan; 5 Graduate School of Biotechnology and Bioengineering, Yuan Ze University, Taoyuan 320315, Taiwan

**Keywords:** carcinoma, ovarian epithelial, intraperitoneal chemotherapy, intraperitoneal, liposomal doxorubicin

## Abstract

**Background/Objectives:** Pegylated liposomal doxorubicin (PLD) has been used in hyperthermic intraperitoneal chemotherapy and early postoperative intraperitoneal chemotherapy. However, the safety and feasibility of repeated intraperitoneal administration of PLD have not been previously reported. This retrospective comparative study aimed to evaluate the feasibility, safety, and preliminary clinical outcomes of repeated intraperitoneal platinum combined with PLD in women with first recurrent ovarian cancer, compared with conventional intravenous platinum/PLD. **Methods**: We retrospectively reviewed all consecutive women with first recurrent ovarian cancer who received either repeated intraperitoneal platinum combined with intraperitoneal and intravenous PLD (IP group, *n* = 13) or intravenous platinum combined with intravenous PLD (IV group, *n* = 17) at a tertiary referral center. **Results:** Median progression-free survival was 13.7 months (95% confidence interval [CI], 10.2 months to not reached) in the IP group and 11.9 months (95% CI, 7.0–14.2 months) in the IV group (*p* = 0.179). Median overall survival was not reached in the IP group and 30.5 months in the IV group (*p* = 0.510). Toxicity profiles were comparable between groups. No cases of peritoneal sclerosis or adhesion-related bowel obstruction were observed following repeated intraperitoneal PLD. **Conclusions**: Repeated intraperitoneal administration of platinum combined with PLD appears to be a safe and feasible treatment strategy for recurrent epithelial ovarian cancer. However, whether this intraperitoneal approach provides additional clinical benefit over conventional intravenous platinum/PLD remains uncertain and warrants further investigation in large-scale studies.

## Simple Summary

Recurrent ovarian cancer remains challenging to treat, particularly when the disease recurs within the peritoneal cavity. Although intravenous platinum-based chemotherapy combined with pegylated liposomal doxorubicin (PLD) is a standard treatment option, the role of intraperitoneal administration of PLD has not been well studied. In this retrospective study, we evaluated 13 patients with first recurrent epithelial ovarian cancer who received repeated intraperitoneal platinum-based chemotherapy combined with intraperitoneal and intravenous PLD therapy, and we compared their outcomes with those of patients receiving intravenous platinum/PLD. Intraperitoneal treatment was generally well tolerated, with a safety profile comparable to that of intravenous therapy. The objective response rate was 85%, and the median progression-free survival was 13.7 months. These preliminary findings suggest that repeated postoperative intraperitoneal PLD is a feasible treatment strategy for selected patients and support further prospective studies to better define its clinical role.

## 1. Introduction

The treatment landscape for recurrent ovarian cancer, fallopian tube cancer, and primary peritoneal cancer has evolved substantially over the past decade. For patients with platinum-sensitive recurrence, platinum-based doublet chemotherapy remains the cornerstone of treatment, while maintenance strategies including poly(ADP-ribose) polymerase inhibitors (PARPis) and antiangiogenic therapy have improved progression-free survival (PFS) in selected populations [1,2,3,4].

However, despite these advances, several challenges remain. In addition to the economic burden associated with these therapies, the effect of adding bevacizumab to platinum-based chemotherapy on overall survival (OS) in recurrent ovarian cancer remains controversial [4,5,6]. Likewise, although olaparib has demonstrated an OS benefit in women with recurrent BRCA1/2-mutated ovarian cancer [1], randomized trials have not consistently shown an OS benefit with PARPis in the broader recurrent ovarian cancer population [7,8]. Therefore, there remains an unmet need for novel treatment strategies to further improve survival outcomes in women with recurrent ovarian cancer.

Intraperitoneal (IP) chemotherapy has demonstrated a survival benefit as part of first-line treatment for advanced ovarian cancer, fallopian tube cancer, and primary peritoneal cancer [9,10,11]. In the recurrent setting, IP administration of several cytotoxic agents, including etoposide, cytarabine, 5-fluorouracil, cisplatin, and paclitaxel, has also been investigated [12,13,14,15,16,17]. Notably, Lu et al. reported that IP chemotherapy with cisplatin or paclitaxel improved PFS in patients with recurrent ovarian cancer compared with intravenous (IV) chemotherapy [17]. Among systemic therapies for platinum-sensitive recurrent ovarian cancer, the CALYPSO trial established IV carboplatin combined with IV pegylated liposomal doxorubicin (PLD) as an effective standard regimen, demonstrating superior PFS and a more favorable toxicity profile than IV carboplatin–paclitaxel [18]. PLD is a formulation of doxorubicin encapsulated in long-circulating liposomes that can significantly alter the pharmacokinetics and biodistribution of the drug [19,20]. Its mechanism of action includes prolonged circulation time, preferential tumor accumulation through enhanced permeability and retention effects, and good pharmacokinetic properties in the peritoneal cavity [21,22,23]. PLD has been used as an IP chemotherapy drug in hyperthermic intraperitoneal chemotherapy (HIPEC) or early postoperative IP chemotherapy [23]. Taken together, these findings provide a strong rationale for investigating repeated IP administration of PLD as a novel treatment strategy for recurrent ovarian cancer.

To our knowledge, the clinical outcomes of repeated IP administration of PLD have not been reported. Therefore, this retrospective study aims to analyze in detail the clinical efficacy and adverse effects of patients with recurrent ovarian cancer who received IP PLD and compare them with those who received IV PLD.

## 2. Materials and Methods

From March 2014 to September 2025, the medical records of all consecutive women with platinum-sensitive first recurrence of ovarian, fallopian tube, or primary peritoneal cancer who received either platinum-based chemotherapy combined with repeated IP PLD (IP group) or conventional IV platinum/PLD (IV group) at a tertiary referral center were retrospectively reviewed. The study protocol was approved by the Institutional Review Board of our institution.

In general, patients receiving chemotherapy for recurrent ovarian cancer at our institution were required to have adequate organ function, including a serum creatinine concentration ≤ 1.3 mg/dL or creatinine clearance ≥ 40 mL/min, total bilirubin ≤ 2 mg/dL, transaminase levels ≤ 3 times the upper limit of normal, left ventricular ejection fraction ≥ 50%, absolute neutrophil count ≥ 1500/μL, platelet count ≥ 100,000/μL, and an Eastern Cooperative Oncology Group (ECOG) performance status of 0–2. Women considered for IP chemotherapy were additionally required to have no extensive peritoneal adhesions that would preclude adequate IP drug distribution.

IP chemotherapy was defined as the direct injection of one or more cycles of chemotherapy drugs into the peritoneal cavity. At our institution, women with platinum-sensitive recurrent ovarian cancer were generally treated with platinum-based combination chemotherapy, including paclitaxel, PLD, or gemcitabine, according to the physician’s discretion and individual patient characteristics. The use of maintenance therapy, including bevacizumab and PARPi, was determined according to platinum sensitivity, BRCA/homologous recombination deficiency (HRD) status, physician discretion, patient preference, financial considerations, and reimbursement availability.

For women receiving IP chemotherapy, a subcutaneous IP access port connected to a flexible catheter was implanted, typically near the lower edge of the breast. Furthermore, if a secondary debulking surgery or laparoscopic IP reservoir and catheter placement had been performed, peritoneal adhesions would be separated as much as possible during the procedure.

The PLD used in this study was distearoylphosphatidylcholine PLD (Lipo-Dox^®^, TTY Biopharm, Taipei, Taiwan), which exhibits pharmacokinetic and toxicological properties characteristic of second-generation PLDs [24,25,26]. In the IP group, the platinum/PLD regimen was administered as follows. In each 28-day cycle, cisplatin 75–100 mg/m^2^, diluted in 1000 mL of normal saline, was infused intraperitoneally on day 1. This was followed by PLD 15 mg/m^2^, diluted in 250 mL of 5% dextrose, administered intravenously over 1 h on day 2, and PLD 15 mg/m^2^, diluted in 250 mL of 5% dextrose, administered intraperitoneally at a free-flow rate on day 2. For patients with an estimated glomerular filtration rate < 50 mL/min/1.73 m^2^, carboplatin was substituted for cisplatin. Carboplatin was dosed using the Calvert formula to achieve a target area under the concentration–time curve (AUC) of 5–6 mg/mL per min [27]. The IV chemotherapy regimen was given as 30–40 mg/m^2^ PLD and carboplatin AUC 5–6. The treatment was repeated every 28 days. Dose modifications were performed according to treatment-related toxicities. Specifically, cisplatin was reduced from 100 mg/m^2^ to 75 mg/m^2^ in patients who developed grade 1 nephrotoxicity and was replaced with carboplatin in those who developed grade 2 nephrotoxicity. PLD dose reduction or discontinuation was uncommon and was reserved for clinically significant PLD-related toxicities, such as hand-foot syndrome or severe mucositis.

If the IP catheter became obstructed during treatment, IP chemotherapy was either converted to IV administration or a laparoscopic IP catheter reimplantation was performed, depending on the clinical situation. Treatment was generally planned for six cycles, with up to eight cycles administered at the discretion of the treating physician based on treatment response, tolerability, and the patient’s clinical condition. The route of PLD administration (IP or IV) was determined by the treating gynecologic oncologist at the initiation of chemotherapy for recurrent disease, based on clinical judgment and patient preference.

Tumor response was evaluated according to the Response Evaluation Criteria in Solid Tumors (RECIST) version 1.1 for patients with measurable disease [28]. For patients without measurable lesions but with serum cancer antigen 125 (CA-125) levels ≥ 40 IU/mL, response was assessed using the Gynecologic Cancer InterGroup (GCIG) CA-125 criteria proposed by Rustin et al. [29,30]. Complete response (CR) was defined as the disappearance of all clinical, radiologic, and biochemical evidence of disease. Partial response (PR) was defined as a ≥30% reduction in the sum of target lesion diameters according to RECIST 1.1, or a ≥50% reduction in CA-125 levels sustained for at least 28 days. Progressive disease (PD) was defined as a ≥20% increase in the sum of target lesion diameters, the appearance of new lesions, or progression according to GCIG CA-125 criteria (i.e., A CA-125 result ≥ 2 × the upper limit of the reference range or ≥ 2 × the nadir on two separate occasions at least one week apart). Stable disease (SD) was defined as neither sufficient tumor shrinkage to qualify for PR nor sufficient increase to qualify for PD [28,29,30]. Treatment-related adverse events were graded according to the Common Terminology Criteria for Adverse Events (CTCAE), version 5.0 [31]. For each patient, the highest-grade toxicity observed during the entire treatment course was recorded and included in the analysis.

The criteria for determining disease recurrence are disease progression as defined in CA-125, the appearance of radiological abnormalities, or histological confirmation by biopsy, whichever occurs first. OS was defined as the interval from initiation of chemotherapy treatment for recurrent disease to death from any cause or the date of last follow-up. PFS was defined as the interval from initiation of chemotherapy treatment for recurrent disease to disease progression, recurrence, death from any cause, or the date of last follow-up, whichever occurred first.

Statistical analyses were performed using Stata version 11.0 (StataCorp LLC, College Station, TX, USA). Multivariable Cox proportional hazards models were performed to identify factors predicting PFS and OS. The multivariable Cox proportional hazards model was restricted to variables with *p* < 0.05 in the univariate analysis, together with treatment modality (IP versus IV), which was included as the primary variable of interest.

Schoenfeld recommended that a sample size of at least 25 patients is needed to achieve the statistical power required to detect specific standardized differences [32]. Julius also suggested including at least 12 subjects in each group in the pilot trial [33]. Therefore, a sample size of at least 12 patients in each group should be adequate to report preliminary clinical results.

## 3. Results

**Figure 1:**
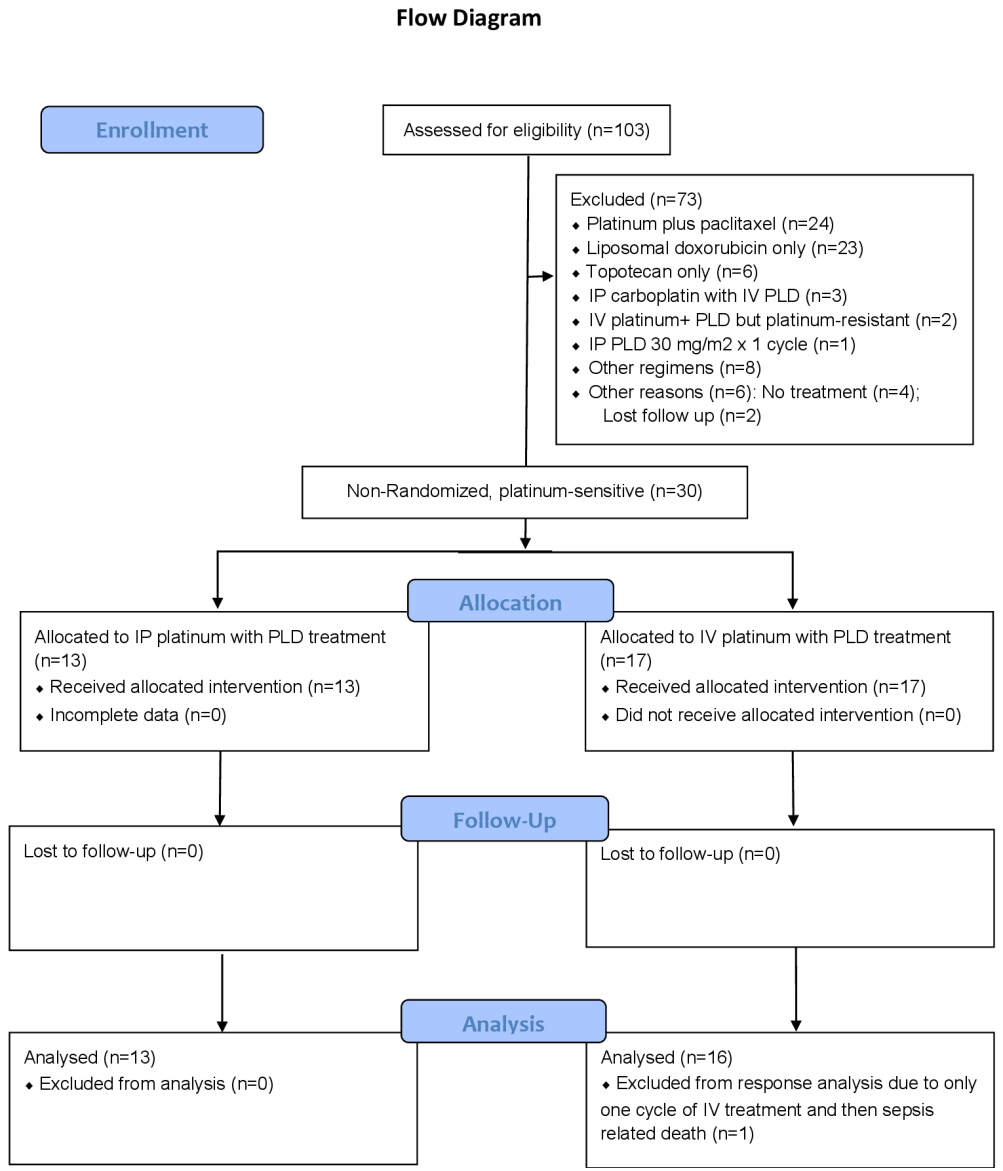
Flowchart of patient selection for women with first recurrent ovarian cancer.

From September 2019 to September 2025, 13 consecutive women received repeated IP PLD (IP group). For comparison, 17 consecutive women who received conventional IV platinum/PLD between March 2014 and September 2025 were identified as the IV group (Figure 1).

Thirteen women received repeated IP platinum-based chemotherapy combined with IP PLD (15 mg/m^2^) and IV PLD (15 mg/m^2^). IP platinum consisted of cisplatin 100 mg/m^2^ (*n* = 7), cisplatin 75 mg/m^2^ (*n* = 2), and carboplatin (*n* = 4). In the IV group, 17 women received IV carboplatin combined with IV PLD. Carboplatin was administered at a target AUC of 6 (*n* = 9), 5 (*n* = 7), or 4 (*n* = 1), while PLD was administered at a dose of 40 mg/m^2^ in 7 women and 30 mg/m^2^ in 10 women. The cumulative PLD dose was comparable between the two groups (164 ± 36 mg in the IP group vs. 192 ± 55 mg in the IV group, *p* = 0.07).

The baseline characteristics of the study population are summarized in Table 1. Apart from differences in frontline treatment regimens, the use of HIPEC at recurrence, and the frequency of secondary cytoreductive surgery, baseline demographic and clinicopathological characteristics were comparable between the IP and IV groups (Table 1).

**Figure 2:**
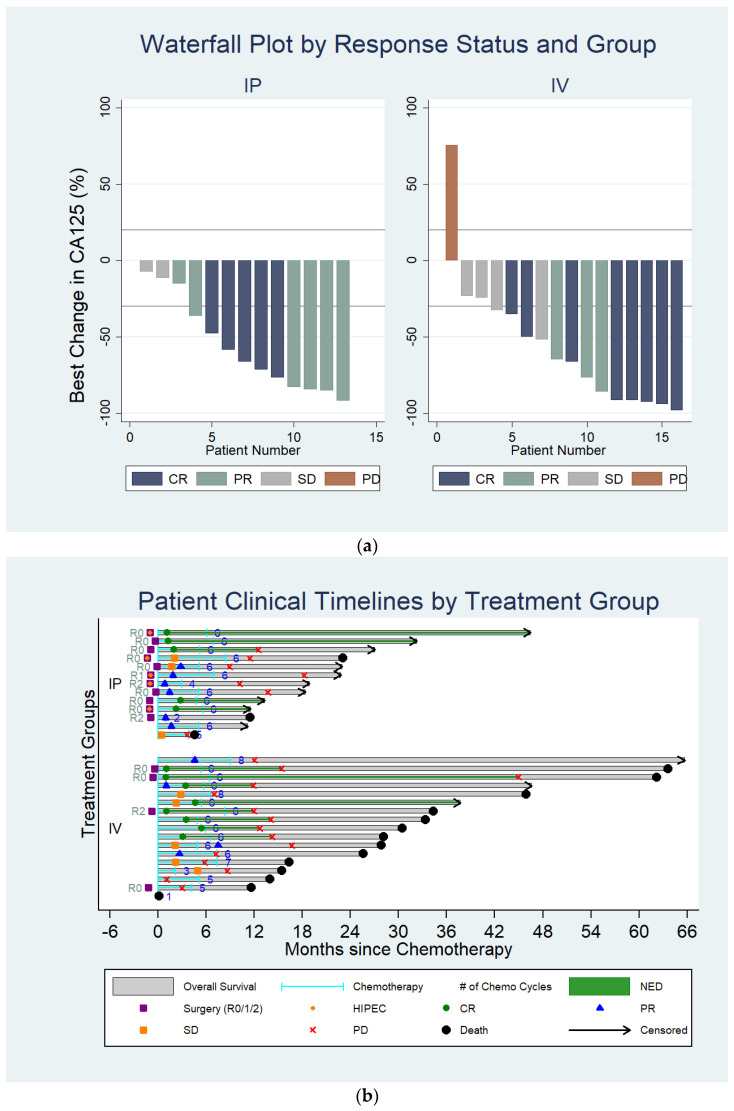
(**a**) The best treatment response and (**b**) clinical course of all women who received intraperitoneal (IP, *n* = 13) or intravenous (IV, *n* = 17) PLD treatment.

The waterfall plot (Figure 2a) illustrates the best treatment response in CA125 for each patient in both groups, whereas the swimmer plot (Figure 2b) depicts the corresponding clinical course and treatment duration for both groups.

**Figure 3:**
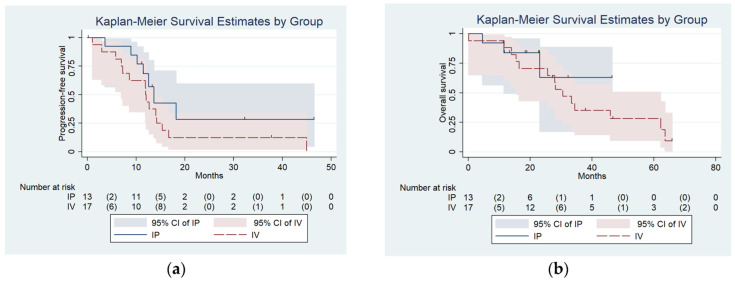
(**a**) Progression-free survival and (**b**) overall survival of all women who received intraperitoneal (IP, *n* = 13) or intravenous (IV, *n* = 17) PLD treatment.

There were no statistically significant differences in PFS or OS between the IP and IV groups (*p* = 0.179 for PFS and *p* = 0.510 for OS; Table 1 and Figure 3a,b). Although the Kaplan–Meier survival curves for PFS showed a separation between the two groups, this difference did not reach statistical significance (Figure 3a).

Among the 13 women in the IP group, 10 (77%) completed the planned six cycles of IP chemotherapy. One woman discontinued IP chemotherapy after four cycles because of IP catheter obstruction and subsequently completed two additional cycles of IV carboplatin (AUC 5) plus PLD (30 mg/m^2^). Another woman discontinued treatment after five cycles because of disease progression. The remaining woman was lost to follow-up after receiving two cycles of IP chemotherapy.

During follow-up, seven women in the IP group developed recurrent disease, three of whom subsequently underwent repeat surgery. Importantly, no cases of peritoneal sclerosis were identified among women who underwent repeat surgery. Likewise, no cases of adhesion-related bowel obstruction were observed in the IP group.

Multivariable Cox proportional hazards analysis did not identify any independent predictors of PFS (Table 2). However, previous dose-dense chemotherapy treatment was identified as a predictor of better OS (HR = 0.086, *p* = 0.04; Table 3).

The most common grade 3 or 4 toxicities were hematologic toxicities: neutropenia (*n* = 5, 36%) and anemia (*n* = 1, 7%) (Table 4). One patient died after receiving a course of IV chemotherapy due to septic shock.

## 4. Discussion

In the present study, treatment-related toxicity profiles were comparable between the IP and IV groups. Importantly, no cases of peritoneal sclerosis or adhesion-related bowel obstruction were identified among women who subsequently underwent repeat surgery. These findings suggest that repeated IP administration of PLD at a dose of 15 mg/m^2^ for up to six cycles seems to be safe, without evidence of clinically significant long-term peritoneal toxicity.

In the CALYPSO trial, in which both carboplatin and PLD were administered intravenously, the median PFS and OS were 11.3 and 30.7 months, respectively [18,34]. In the present study, the median PFS was 13.7 months in the IP group, whereas the median OS was not reached during follow-up. However, neither PFS nor OS differed significantly between the IP and IV groups (Table 1). Although the Kaplan–Meier survival curves for PFS showed a separation between the two groups, this difference did not reach statistical significance (Figure 3a). Therefore, repeated IP PLD appears to be a feasible treatment strategy for selected women with platinum-sensitive recurrent ovarian cancer, although its comparative efficacy relative to conventional IV PLD remains uncertain and requires confirmation in larger prospective studies.

Several studies have demonstrated the potential role of IP chemotherapy in recurrent ovarian cancer [12,13,14,15,16,17]. Lu et al. found a potential survival benefit associated with IP chemotherapy compared with conventional IV chemotherapy in recurrent disease, possibly due to enhanced local drug delivery [17]. Similarly, the CHIPOR trial demonstrated an OS benefit associated with HIPEC in selected women undergoing secondary cytoreductive surgery for platinum-sensitive recurrent ovarian cancer [35]. However, HIPEC is limited to a single intraoperative treatment session. In contrast, repeated IP chemotherapy permits multiple treatment cycles and sustained regional drug exposure. Although the present study was not designed to directly compare these treatment strategies, our findings seem to support that repeated IP PLD can be safely administered over multiple treatment cycles without evidence of clinically significant long-term peritoneal toxicity.

PLD, in particular, has been shown to preferentially accumulate in tumor tissue due to enhanced permeability of tumor vasculature [36]. Furthermore, Sugarbaker emphasized the importance of repeated bidirectional therapy (IP plus systemic chemotherapy) in improving treatment outcomes [36], as in the IP group of this study. From a pharmacologic perspective, combining IP and IV PLD may therefore provide both regional and systemic drug delivery. The recommended IV dose of PLD in combination with carboplatin for platinum-sensitive recurrent ovarian cancer is 30 mg/m^2^ [23]. In a pharmacokinetic study of IP PLD administered during HIPEC, approximately 40% of the administered dose was absorbed during the 180 min perfusion period, with cumulative systemic absorption approaching 80% within 24 h [23]. Based on these pharmacokinetic observations, we selected a bidirectional regimen consisting of 15 mg/m^2^ administered intraperitoneally and 15 mg/m^2^ administered intravenously. Assuming comparable systemic absorption of PLD following postoperative intraperitoneal administration, the estimated systemic equivalent dose of PLD would be approximately 27 mg/m^2^ (15 mg/m^2^ × 80% + 15 mg/m^2^), which closely approximates the standard intravenous dose (i.e., 30 mg/m^2^). Although this calculation is theoretical and requires pharmacokinetic validation, it provides a biologic rationale for the dose selection used in the present study.

In addition to PARPis, antiangiogenic agents [1,2,3,4], and IP chemotherapy, the therapeutic landscape of recurrent ovarian cancer continues to evolve. Several novel treatment strategies, particularly for platinum-resistant disease, have recently demonstrated encouraging clinical activity. These include immune checkpoint inhibition with pembrolizumab plus paclitaxel, which improved OS compared with placebo plus paclitaxel (HR = 0.82, *p* = 0.011) [37]; the selective glucocorticoid receptor antagonist relacorilant combined with nab-paclitaxel, which significantly prolonged OS compared with nab-paclitaxel alone (HR = 0.65, *p* = 0.0004) [38]; the folate receptor-α–targeted antibody–drug conjugate mirvetuximab soravtansine, which demonstrated superior OS compared with investigator’s-choice chemotherapy in patients with folate receptor-α-positive ovarian cancer (HR = 0.67, *p* = 0.005) [39]; and HER2-targeted antibody–drug conjugates, which have shown promising antitumor activity in HER2-expressing ovarian cancers [40]. These advances highlight the increasing importance of biomarker-directed therapies in recurrent ovarian cancer. Nevertheless, platinum-based chemotherapy remains the standard treatment for women with platinum-sensitive recurrence, underscoring the need to further optimize platinum-based treatment strategies, including intraperitoneal drug delivery.

This study has several limitations. First, the retrospective, nonrandomized design, single-center, limited sample size, and treatment allocation based on physician discretion might introduce the potential for selection bias. Secondly, the higher incidence of secondary cytoreductive surgery and HIPEC in the IP group may have also biased the results. In addition, the higher proportion of complete resection in women may have helped to observe a good prognosis in the IP group. Finally, differences in follow-up duration between groups may also have influenced survival estimates. Therefore, future large-scale studies are warranted to better define the role of repeated IP PLD in platinum-sensitive recurrent ovarian cancer.

## 5. Conclusions

Repeated IP administration of platinum combined with PLD seems to be a safe and feasible treatment strategy for the treatment of platinum-sensitive recurrent epithelial ovarian cancer. However, whether this approach provides additional clinical benefit over conventional IV platinum/PLD remains uncertain and warrants further investigation in large-scale studies.

## Figures and Tables

**Table 1:** Baseline characteristics of women with recurrent ovarian, fallopian tube, or primary peritoneal cancer (*n* = 30).

Variable	IP (*n* = 13)	IV (*n* = 17)	^a^ *p*
Age (years)	60 (47, 63)	59 (54, 64.5)	0.818
Body mass index (kg/m^2^)	22.9 (19.5, 27.1)	24.6 (22.4, 26.2)	0.215
Baseline CA-125 (U/mL)	916 (243, 2029)	1046 (383, 2265)	0.630
CA-125 at recurrence (U/mL)	91 (47, 114)	92 (62, 165)	0.676
ECOG Score			
0	1 (8)	4 (24)	0.436
1	9 (69)	7 (41)	
2	3 (23)	5 (29)	
3	0 (0)	1 (6)	
FIGO Stage			
I	2 (15)	1 (6)	0.249
II	1 (8)	0 (0)	
III	9 (69)	10 (59)	
IV	1 (8)	6 (35)	
Histologic Subtype			
Serous	9 (69)	12 (71)	0.720
Clear cell	2 (15)	1 (6)	
Miscellaneous	2 (15)	4 (24)	
Genetic Status			
BRCA 1/2	1 (8)	1 (6)	0.736
HRD	2 (15)	0 (0)	
Wild type	7 (49)	5 (29)	
First-Line Chemotherapy Regimen			0.030
Triweekly IV carboplatin plus paclitaxel	4 (31)	8 (47)	
IP platinum plus paclitaxel	8 (62)	3 (18)	
Dose-dense carboplatin plus paclitaxel	0 (0)	5 (29)	
Triweekly IV platinum plus cyclophosphamide	1 (8)	1 (6)	
Progression-free survival after first-line treatment (months)	27.5 (9.6, 48.3)	10.6 (8.1, 14.8)	0.052
Ascites at recurrence	3 (23)	6 (35)	0.691
HIPEC at recurrence	5 (38)	0 (0)	0.009
Bevacizumab use at recurrence	3 (23)	4 (24)	1.000
PARPi use at recurrence	2 (15)	1 (6)	0.565
2nd debulking surgery	11 (85)	5 (29)	0.004
Residual Disease Status at 2nd Debulking Surgery			
No residual disease	8 (58)	3 (18)	0.698
0 < diameter ≤ 1 cm	1 (8)	0 (0)	
Diameter >1 cm	2 (17)	2 (12)	
Cycles of PLD	6 (5.5, 6)	6 (6, 6)	0.469
Clinical Response			
CR	5 (38)	8 (50)	0.450
PR	6 (38)	3 (19)	
SD	2(15)	4 (25)	
PD	0 (8)	1 (6)	
Response duration	10.5 (7.7, 14.3)	9.2 (4.6, 11.1)	0.442
Overall response rate	11 (85)	11 (69)	0.292
Disease control rate	13 (100)	15 (94)	1.000
Progression/recurrence	7 (54)	15 (88)	-
Death	3 (23)	14 (82)	-
Progression-free survival (months)	13.7 (10.2 to infinity)	11.9 (7.0 to 14.2)	0.179
Overall survival (months)	Not reached (11.5 to infinity)	30.5 (15.5 to 62.2)	0.510
Follow-up interval (months)	20.3 (13.7 to 26.9)	30.5 (15.9 to 46.3)	0.031

Values are expressed as median (interquartile range), number (percentage), or median (95% confidence interval). CA-125 = cancer antigen 125, CR = complete response, ECOG = Eastern Cooperative Oncology Group, FIGO = The International Federation of Gynecology and Obstetrics, HIPEC = hyperthermic intraperitoneal chemotherapy, HRD= homologous recombination deficiency, IP = intraperitoneal, IV = intravenous, PARPi = poly(ADP-ribose) polymerase inhibitors, PD = progressive disease, PLD = pegylated liposomal doxorubicin, PR = partial response, SD = stable disease. ^a^ By Wilcoxon rank-sum test, chi-square test, Fisher’s exact test, or log-rank test. Note: One patient in the IV group died of septic shock on the third day after receiving the first cycle of chemotherapy. Therefore, this patient was excluded from efficacy evaluation.

**Table 2:** Cox proportional hazards model to predict progression-free survival (*n* = 30).

		Univariate			Multivariable	
	Hazard Ratio	95% CI	^a^ *p*	Hazard Ratio	95% CI	^b^ *p*
IP PLD	0.543	0.220 to 1.340	0.185	1.111	0.224 to 5.524	0.897
First-line chemotherapy regimen						
Triweekly IV carboplatin plus paclitaxel	1.000	-	-	1.000	-	-
IP platinum plus paclitaxel	0.791	0.290 to 2.157	0.647	0.880	0.320 to 2.243	0.805
Dose-dense carboplatin plus paclitaxel	0.831	0.254 to 2.720	0.759	1.071	0.236 to 4.867	0.929
IV platinum plus cyclophosphamide	2.267	0.454 to 11.329	0.319	4.302	0.560 to 44.047	0.161
HIPEC at the first recurrence	0.532	0.154 to 1.835	0.318	0.920	0.182 to 4.659	0.920
2nd debulking surgery	0.489	0.204 to 1.171	0.108	0.381	0.080 to 1.820	0.226

CI = confidence interval, HIPEC = hyperthermic intraperitoneal chemotherapy, IP = intraperitoneal, IV = intravenous, PLD = pegylated liposomal doxorubicin. ^a^ Univariate Cox proportional hazards modeling. ^b^ Multivariable Cox proportional hazards modeling was performed using all variables with *p* < 0.05 in the univariate analysis.

**Table 3:** Cox proportional hazards model to predict overall survival (*n* = 30).

		Univariate			Multivariable	
	Hazard Ratio	95% CI	^a^ *p*	Hazard Ratio	95% CI	^b^ *p*
IP PLD	0.648	0.376 to 2.380	0.513	0.529	0.060 to 4.662	0.567
First-line chemotherapy regimen						
Triweekly IV carboplatin plus paclitaxel	1.000	-	-	1.000	-	-
IP platinum plus paclitaxel	0.680	0.203 to 2.279	0.532	0.721	0.213 to 2.438	0.599
Dose-dense carboplatin plus paclitaxel	0.131	0.016 to 1.069	0.058	0.086	0.008 to 0.889	0.040
IV platinum plus cyclophosphamide	0.526	0.065 to 4.234	0.546	0.345	0.026 to 4.609	0.421
HIPEC at the first recurrence	0.441	0.058 to 3.377	0.430	0.361	0.031 to 4.252	0.418
2nd debulking surgery	0.567	0.208 to 1.544	0.267	1.377	0.229 to 8.292	0.727

CI = confidence interval, HIPEC = hyperthermic intraperitoneal chemotherapy, IP = intraperitoneal, IV = intravenous, PLD = pegylated liposomal doxorubicin. ^a^ Univariate Cox proportional hazards modeling. ^b^ Multivariable Cox proportional hazards modeling was performed using all variables with *p* < 0.05 in the univariate analysis.

**Table 4:** Treatment-related toxicity profile in women with recurrent ovarian, fallopian tube or primary peritoneal cancer (*n* = 31).

	IP (*n* = 14)				IV (*n* = 17)				
Grade	1	2	3	4	1	2	3	4	^a^ *p*
Hematological									
Neutropenia	4 (29)	3 (21)	5 (36)	0 (0)	2 (12)	1 (6)	10 (58)	3 (18)	0.206
Thrombocytopenia	6 (43)	1 (7)	0 (0)	0 (0)	6 (35)	3 (18)	2 (12)	3 (18)	0.134
Anemia	4 (29)	9 (64)	1 (7)	0 (0)	6 (35)	9 (53)	2 (16)	0 (0)	0.875
Infection	0 (0)	0 (0)	0 (0)	0 (0)	0 (0)	0 (0)	0 (0)	1 (0)	1.000
Non-hematological									
Nausea/vomiting	13 (93)	0 (0)	0 (0)	0 (0)	14 (82)	0 (0)	0 (0)	0 (0)	0.607
Diarrhea	1 (7)	0 (0)	0 (0)	0 (0)	2 (12)	0 (0)	0 (0)	0 (0)	1.000
Stomatitis	8 (57)	0 (0)	0 (0)	0 (0)	7 (41)	0 (0)	0 (0)	0 (0)	0.479
Nephrotoxicity	3 (21)	0 (0)	0 (0)	0 (0)	6 (35)	0 (0)	0 (0)	0 (0)	0.456
Hepatotoxicity	4 (29)	0 (0)	0 (0)	0 (0)	8 (47)	1 (6)	0 (0)	0 (0)	0.275
Constipation	13 (93)	0 (0)	0 (0)	0 (0)	9 (53)	0 (0)	0 (0)	0 (0)	0.021
Cardiotoxicity	0 (0)	0 (0)	0 (0)	0 (0)	1 (6)	0 (0)	0 (0)	0 (0)	1.000
Port-related complications	0 (0)	0 (0)	0 (0)	0 (0)	-	-	-	-	-

Values are expressed as a number (percentage). IP = intraperitoneal, IV = intravenous. ^a^ By chi-square test or Fisher’s exact test.

## Data Availability

The data presented in this study are available on request from the corresponding author. The data are not publicly available due to ethical issues.

## Abbreviations

The following abbreviations are used in this manuscript:

AUC	Area under the curve
CA-125	Cancer antigen 125
CI	Confidence interval
CGIG	Gynecologic Cancer InterGroup
CR	Complete response
CTCAE	Common Terminology Criteria for Adverse Events
eGFR	Estimated glomerular filtration rate
HIPEC	Hyperthermic intraperitoneal chemotherapy
HR	Hazard ratio
HRD	Homologous recombination deficiency
IP	Intraperitoneal
IV	Intravenous
OS	Overall survival
PARPi	Poly(ADP-ribose) polymerase inhibitor
PD	Progressive disease
PFS	Progression-free survival
PLD	Pegylated liposomal doxorubicin
PR	Partial response
RECIST	Response Evaluation Criteria in Solid Tumors
SD	Stable disease
